# Cyclophosphamide in alveolar hemorrahge due to leptospirosis

**DOI:** 10.4103/0972-5229.68231

**Published:** 2010

**Authors:** Anand V. Joshi, Farhad Kapadia

**Affiliations:** Department of Critical Care Medicine, PD Hinduja National Hospital, Mumbai, India

Dear Editor,

We read with interest the research article Cyclophosphamide in pulmonary alveolar hemorrahage due to leptospirosis by Trivedi *et al*.[1] We would like to have some clarification regarding the design and findings of the study.

Regarding the methodology of the study, we have a comment and some questions. We disagree with the authors’ statement, “in view of the life-threatening nature of the disease, a randomized trial was not possible”. We are unclear if this is a prospective observational series with historical controls or a non-randomized controlled trial with contemporary controls. As we understand it, a high mortality of 29/32 was noted in the initial group of patients, and cyclophosphamide was then used in the subsequent 33 patients (of whom 22 survived). Could the authors clarify if they submitted the protocol and got clearance from the Human Research Ethics Committee before the 32 control and 33 study patients, or after the 32 control but before the 33 study patients were enrolled.

We would like the authors to clarify if they used cyclophosphamide only if there was clear evidence of pulmonary alveolar hemorrhage, or if they used it for all patients with severe pulmonary involvement in leptospirosis. A Murray ALI score of ≥ 2.5 could be due to alveolar hemorrhage or due to non hemorrhagic conditions like ARDS, pulmonary edema, atelectasis and secondary bacterial pneumonia. Could the authors specify which criteria they used for identifying the subgroup of those with pulmonary alveolar hemorrhage from patients with other forms of pulmonary complications secondary to leptospirosis.

We would also like to authors to give us information regarding the severity of illness and the need of standard critical care in these patients. We note that NIV was used in all patients. We would like further information as to how may in each group got intubated, how many needed invasive hemodynamic monitoring and inotropes or pressors. We note that none received renal replacement therapy. Could the authors give more information about this considering that there was an 80% renal involvement in both groups, and the overall mortality was 40/65. We would also appreciate it if any of the authors could give us a more definite end point, like 28 day mortality, rather than the follow-up only till the counts returned to normal or the patient was discharged from hospital.

We feel that the concept of using cyclophosphamide for alveolar hemorrhage in leptospirosis has potential but would like more complete data before we start using this promising approach.
